# Selective Attenuation of Norepinephrine Release and Stress-Induced Heart Rate Increase by Partial Adenosine A_1_ Agonism

**DOI:** 10.1371/journal.pone.0018048

**Published:** 2011-03-28

**Authors:** Lorenz Bott-Flügel, Alexandra Bernshausen, Heike Schneider, Peter Luppa, Katja Zimmermann, Barbara Albrecht-Küpper, Raimund Kast, Karl-Ludwig Laugwitz, Heimo Ehmke, Andreas Knorr, Melchior Seyfarth

**Affiliations:** 1 1. Medizinische Klinik, Klinikum rechts der Isar and Deutsches Herzzentrum München, Technische Universität, München, Germany; 2 Institut für Klinische Chemie und Pathobiochemie, Klinikum rechts der Isar, Technische Universität, München, Germany; 3 Bayer Schering Pharma AG, Global Drug Discovery, Wuppertal, Germany; 4 Institut für Vegetative Physiologie und Pathophysiologie, Universitätsklinikum Hamburg-Eppendorf, Hamburg, Germany; 5 Medizinische Klinik 3, HELIOS Klinikum Wuppertal and Lehrstuhl für Kardiologie, Universität Witten/Herdecke, Witten, Germany; Universitätsklinikum Schleswig-Holstein - Campus Luebeck, Germany

## Abstract

The release of the neurotransmitter norepinephrine (NE) is modulated by presynaptic adenosine receptors. In the present study we investigated the effect of a partial activation of this feedback mechanism. We hypothesized that partial agonism would have differential effects on NE release in isolated hearts as well as on heart rate *in vivo* depending on the genetic background and baseline sympathetic activity. In isolated perfused hearts of Wistar and Spontaneously Hypertensive Rats (SHR), NE release was induced by electrical stimulation under control conditions (S1), and with capadenoson 6 · 10^−8^ M (30 µg/l), 6 · 10^−7^ M (300 µg/l) or 2-chloro-N^6^-cyclopentyladenosine (CCPA) 10^−6^ M (S2). Under control conditions (S1), NE release was significantly higher in SHR hearts compared to Wistar (766+/−87 pmol/g vs. 173+/−18 pmol/g, p<0.01). Capadenoson led to a concentration-dependent decrease of the stimulation–induced NE release in SHR (S2/S1 = 0.90±0.08 with capadenoson 6 · 10^−8^ M, 0.54±0.02 with 6 · 10^−7^ M), but not in Wistar hearts (S2/S1 = 1.05±0.12 with 6 · 10^−8^ M, 1.03±0.09 with 6 · 10^−7^ M). CCPA reduced NE release to a similar degree in hearts from both strains. *In vivo* capadenoson did not alter resting heart rate in Wistar rats or SHR. Restraint stress induced a significantly greater increase of heart rate in SHR than in Wistar rats. Capadenoson blunted this stress-induced tachycardia by 45% in SHR, but not in Wistar rats. Using a [^35^S]GTPγS assay we demonstrated that capadenoson is a partial agonist compared to the full agonist CCPA (74+/−2% A_1_-receptor stimulation). These results suggest that partial adenosine A_1_-agonism dampens stress-induced tachycardia selectively in rats susceptible to strong increases in sympathetic activity, most likely due to a presynaptic attenuation of NE release.

## Introduction

Sympathetic activity regulates heart rate and cardiac function via stimulation of a wide variety of G-protein coupled receptors, such as alpha- and beta-adrenergic receptors [1]. The only class of drugs to date to be widely used to modulate the activity of the sympathetic nervous system are beta blockers, mostly β_1_-selective, which act by postsynaptic blockade of beta-adrenoceptors in the heart. They do not influence presynaptic regulation of sympathetic activity and the release of the neurotransmitter norepinephrine (NE) [2], [3].

A different way of modulating sympathetic activity is the modulation of the release of NE from pre- or postganglionic nerve terminals. Moxonidine, for example, is thought to attenuate central sympathetic tone either by stimulating central I_1_-imidazoline receptors in the medulla oblongata [4], [5], [6], or via central presynaptic alpha-adrenoceptors [7], thereby reducing sympathetic tone in the central nervous system. Clonidine, a central α_2_-receptor agonist, also inhibits sympathetic tone by peripheral presynaptic inhibition of transmitter release from postganglionic neurons [8]. In the heart, NE is released from sympathetic nerve terminals upon stimulation and acts on the myocardium by modulating heart rate, myocardial contractility and calcium handling via alpha- and beta-adrenergic receptors [9]. Its exocytotic release is presynaptically modulated during myocardial ischemia via adenosine-A_1_ and adrenergic α_2_-receptors [10], [11]. Recently, agonists of the adenosine A_1_ receptor, either full or partial, have been tested in a variety of disease conditions [12]. Because of the potential modulation of NE release in the heart, agonists of the adenosine A_1_ receptor might offer a unique opportunity to selectively modulate sympathetic activity to the heart. In a recent phase-II study in patients with angina pectoris it was noted that the novel A_1_-agonist capadenoson (BAY 68-4986) selectively blunted the heart rate increase during treadmill exercise without altering baseline frequency [12], [13].

This effect of capadenoson on the heart rate could be due to a presynaptic modulation of the release of NE which is particularly effective under conditions of an increased sympathetic nerve activity. To test this hypothesis, the effects of the adenosine-A_1_ agonist capadenoson on the NE release *in vitro* as well as on heart rate *in vivo* was evaluated in two rat strains with genetically different levels of sympathetic activity, Wistar and Spontaneously Hypertensive rats (SHR). The latter strain has an increased sympathetic tone already at rest and exhibits a much stronger increase in sympathetic tone upon restraint stress than Wistar rats [14], [15].

## Methods

### Study drug

Capadenoson (molecular weight 520.0 g/mol) was provided by Bayer Schering Pharma AG (Wuppertal, Germany) as a research compound Bay 68-4986-EXTRUDAT Fa.20 Pt.041027 [16]. This water-soluble formulation contains 5% active substance by weight. For the *in vitro* experiments the substance was solubilised in dimethyl sulfoxide (DMSO) 10%, polyethylene glycol 30%, and physiologic (0.9%) sodium chloride 60% (all Sigma-Aldrich, Germany). The final concentration of DMSO in the perfusate did not exceed 0.01%. For the *in vivo* experiments Bay 68-4986-EXTRUDAT was weighed according to body weight and administered orally. A dose of 0.15 mg/kg was chosen on the basis of previous experiments which showed it not to induce bradycardia in Wistar rats. The formulation was prepared freshly each day before administration.

### Stimulation-induced norepinephrine release

All animal experiments were conducted according to the Guide for the Care and Use of Laboratory Animals (NIH publication No. 86-23, revised 1985), and were approved by the Regierung von Oberbayern (Az 55.2-1-54-2531.3-15-07).

A total of 14 Wistar rats and 18 SHR (Charles River Inc., Germany; body weight 200–50 g, all female) underwent experiments to evaluate the exocytotic, stimulation-induced NE release during electrical field stimulation as described before [11]. Rats were killed by an injection of pentobarbital i.p. (0.5 ml/100 mg body weight, Merial, Germany), and hearts were rapidly excised, and placed in ice cold Krebs-Henseleit solution (KHL). They were quickly mounted on a Langendorff apparatus for retrograde perfusion with KHL. Perfusion rate was kept constant at 10 ml/min, the temperature was adjusted to 37°C, and the pH to 7.4 through bubbling with 5% CO_2_/95% O_2_. Via an inflow line desipramine (Sigma-Aldrich, Germany) at a concentration of 10^−7^ M was added to the perfusion buffer. After an equilibration period of 20 minutes, electrical field stimulation was commenced via two metal paddles adjacent to both sides of the beating heart for 1 minute (5V, 6 Hz). We collected the efflux in plastic tubes the minute before, during, and 3 minutes after the stimulation. These were rapidly frozen in liquid nitrogen and stored at −20°C till analysis. The NE release was calculated as the cumulative release induced by the electrical stimulation. After the first stimulation (S1), the study drug capadenoson at concentrations of 30 µg/l (6 · 10^−8^ M) or 300 µg/l (6 · 10^−7^ M), or 2-chloro-N^6^-cyclopentyladenosine (CCPA, 10^−6^ M, Sigma-Aldrich), respectively, were added via separate perfusion lines for 30 minutes. After this time a second stimulation (S2) was executed to determine the effect of the drugs on NE release compared to the first stimulation. The effect of each pharmacological intervention was analysed by calculating the ratio of NE release induced by the second and first stimulation (S2/S1 ratio).

### Determination of norepinephrine concentration

Norepinephrine was detected in the samples using high-performance liquid chromatography (HPLC) with electrochemical detection (ECD) [17]. After thawing samples were prepared by a reagent kit for “catecholamines in plasma” (Chromsystems, Germany). Shortly, 1 ml of the sample was mixed with 500 µl extraction buffer plus 50 µl internal standard (IS), and extracted by solid phase extraction (SPE). Elution from SPE column was carried out by 120 µl elution buffer. Fifty µl of the eluted sample were separated by HPLC. Epinephrine and norepinephrine were detected by ECD, and quantified by an external standard involving the IS. The sensitivity of this assay is <15 pg/ml norepinephrine. Values are given as pmol per g wet heart weight.

### Restraint stress experiments and in-vivo telemetry

The in vivo experiments were approved by Landesamt für Natur, Umwelt und Verbraucherschutz (Az 8.87-50.10.44.08.068). Female Wistar rats (n = 24) or SHR (n = 40) at a body weight of 200–220 g (breeder: Charles River, Germany) were included for a restraint stress test [18]. All animals were housed at an ambient temperature of ∼22°C, maintained on a 12-hour light/dark cycle with free access to standard laboratory rat chow and water ad libitum.

Animals were equipped with implantable radiotelemetry, and a data acquisition system (Data Sciences International, St. Paul MN, U.S.A.), comprised of a chronically implantable transducer/transmitter unit equipped with a fluid-filled catheter. Rats were instrumented at least one month prior to the experiments. The transmitter was implanted into the peritoneal cavity and the sensing catheter was inserted into the descending aorta. The animals were allocated to 4 groups:

1) Wistar rats with vehicle or 2) capadenoson 0.15 mg/kg (each n = 12)

3) Hypertensive SHR with vehicle or 4) capadenoson 0.15 mg/kg (each n = 20)

All animals were treated with vehicle or capadenoson for 5 days. Drug administration took place at 9.00 a.m. On day 5, all animals were placed into transparent restraint tubes for a period of 2 hr between 11:00 and 13:00. All animals experienced this treatment for the first time in their life. A dose of 0.15 mg/kg was chosen after prior dose-finding studies, which ruled out significant effects of capadenoson on blood pressure between 0.1 mg/kg and 0.3 mg/kg, whereas there was a significant reduction in resting blood pressure at higher doses (Fig. S1).

### [^35^S]-GTPγS binding assay

Human frontal cortex was obtained from Analytical Biological Services Inc. (Wilmington, DE, USA). Membranes from the human cortex were prepared as described previously [19]. [^35^S]GTPγS binding was measured as described by Lorenzen [20]. Briefly, 5 µg of membrane protein was incubated in a total volume of 160 µl for 2 hr at 25°C in a shaking water bath. [^35^S]GTPγS binding in control incubations and in the presence of capadenoson showed a linear time course up to this incubation time. Binding buffer contained 50 mM Tris/HCl, pH 7.4, 2 mM triethanolamine, 1 mM EDTA, 5 mM MgCI_2_, 10 µM GDP, 1 mM dithiothreitol, 100 mM NaCl, 0.2 units/ml adenosine deaminase, 0.2 nM [^35^S]GTPγS, and 0.5% bovine serum albumin. Non-specific binding was determined in the presence of 10 µM GTPγS. Incubations were terminated through filtration of the samples over multiscreen FB glass fiber filters (Millipore, Billerica, MA, USA) followed by two washes with binding buffer. The filters were dried, coated with scintillator and counted for radioactivity. Binding curves of [^35^S]GTPγS were analyzed by nonlinear regression using GraphPad Prism (GraphPad Software Inc., San Diego, CA, USA).

### GTP shift assay

Preparation of brain membrane: Crude synaptosomal membranes from rat brain were prepared according to the method described by Lohse [21]. Male Wistar rats (150–250 g) were killed be cervical dislocation, the brain cortex was quickly removed and immediately placed in 0.32 mol/l sucrose (4°C). The tissue was homogenized in 10 volumes sucrose in a glass/teflon homogenizer (clearing 0.2 mm; 500 rpm for 30 s). The homogenate was centrifuged at 1000 x g for 10 min to remove the nuclear fraction and the supernatant centrifuged at 30000 x g for 30 min to give the P2 fraction. The pellets were resuspended in 10 ml of water and left on ice for 30 min to give synaptosomal membranes. After a final centrifugation step at 48000 x g for 10 min the membranes were resuspended in 50 mmol/l Tris-HCl buffer, pH 7.4 and incubated with 2 U/ml adenosine deaminase (ADA) at 37°C for 30 min. The membranes were frozen in small aliquots and stored at −80°C until binding assay. Protein was measured according to Bradford, using a BIOrad assay kit.

Receptor binding studies: The adenosine A1 receptor binding assay was carried out on rat brain cortical membranes and with 0.4 nM [^3^H] 8-cyclopentyl-1,3-dipropylxanthine (DPCPX, Kd = 0.28 nM) as the radioligand. The assay was performed as originally described by Lohse et al. [22] and modified by van der Wenden et al. [23]. 10 µg of membrane protein were incubated at 37°C for 20 min with 0.4 nM [^3^H]DPCPX and adenosine A1 agonist in different concentrations in buffer (50 mM Tris-HCl, pH 7.4, 2 U/ml ADA) in the presence and absence of GTP. Nonspecific binding was determined in the presence of 10 µM R-PIA. Incubation was terminated by rapid filtration through GF/B glass fibre filter plates. Filters were washed three times with icecold Tris-HCl buffer 50 mM, pH 7.4. Radioactivity of the filter plate was counted with 100 µl scintillation cocktail in a Microbeta TriLux beta counter (PerkinElmer, Massachusetts, USA).

### Statistical analysis

All values are expressed as mean +/− standard error of means, unless otherwise indicated. Means were compared with ANOVA, unless a nonparametric distribution was assumed. In this case Kruskal-Wallis-test was performed. For the comparison of norepinephrine values from second to first stimulation a paired t-test was used. A p-value <0.05 was considered statistically significant.

## Results

### Effect of capadenoson on stimulation-induced NE release

The effect of capadenoson on stimulation-induced NE release was evaluated in isolated perfused rat hearts of Wistar and SHR strains.

**Figure 1:**
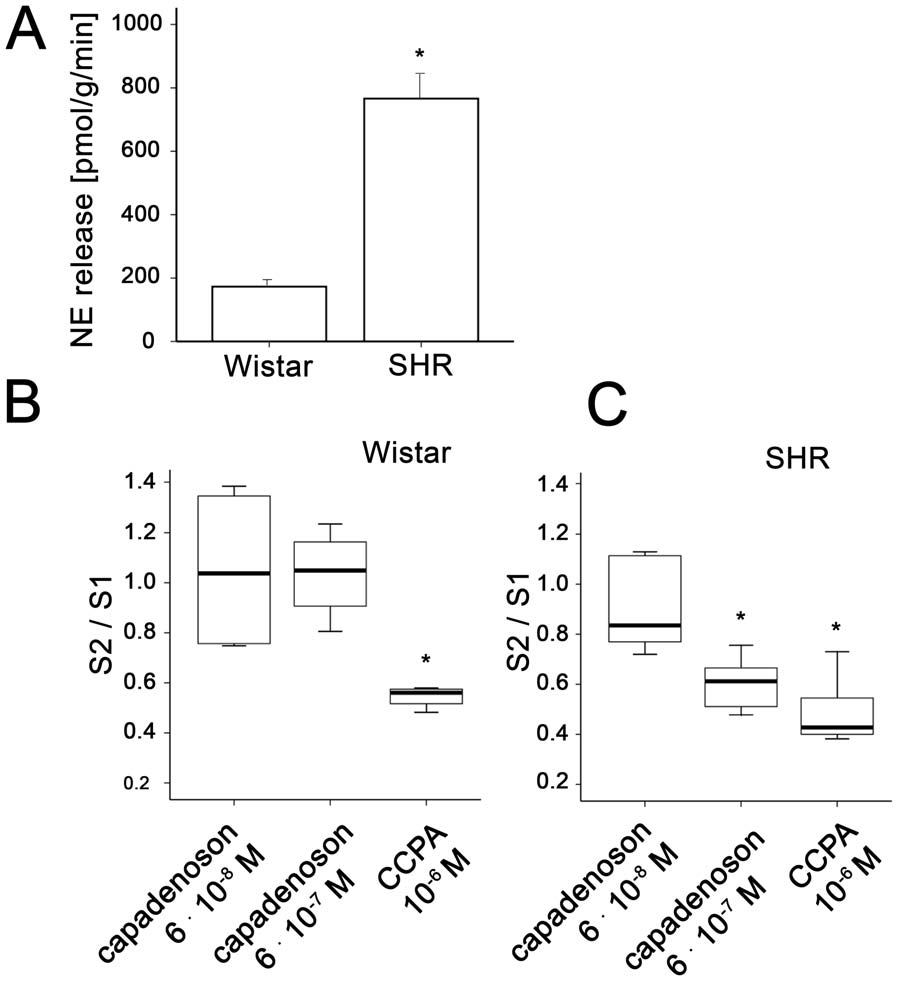
Norepinephrine release from isolated rat hearts is modulated by adenosine agonists. A) Mean of absolute NE concentration released during electrical field stimulation without pharmacologic modulation (Wistar n = 18, SHR n = 14; p<0.01). B) The effect of capadenoson and CCPA (capadenoson 6 · 10^−8^ M n = 6, capadenoson 6 · 10^−7^ M n = 4, CCPA 10^−6^ M n = 4) in Wistar rats. C) The effect of capadenoson and CCPA (capadenoson 6 · 10^−8^ M n = 6, capadenoson 6 · 10^−7^ M n = 5, CCPA n = 7) in SHR. The first stimulation (S1) served as an individual control without intervention; the second stimulation (S2) was performed after 30 minutes pretreatment with either capadenoson or CCPA. S2/S1 expressed as means +/− SEM, * p<0.01 for NE release with pharmacological modulation vs NE release without.

Under basal conditions without pharmacological modulation of A_1_ receptors, the NE release was 4-times higher in SHR compared to Wistar rats (SHR 766+/−87 pmol/g, n = 18, vs. 173+/−18 pmol/g, n = 14; p<0.01, fig. 1A). There was no indication for depletion of norepinephrine under control conditions without pharmacological intervention (Fig. S2).

As demonstrated in Fig. 1B, capadenoson had no effect on NE release in Wistar rats, whereas addition of CCPA at a concentration of 10^−6^ M led to a robust decrease in the amount of NE release (S2/S1 = 1.1+/−0.11 for capadenoson 6 · 10^−8^ M, n = 6, p = 0.9; S2/S1 = 1.0+/−0.09 for capadenoson 6 · 10^−7^ M, n = 4, p = 0.8; S2/S1 = 0.5+/−0.02 for CCPA 10^−6^ M, n = 4, p = 0.007).

To the contrary, in SHR, capadenoson led to a significant concentration-dependent decrease of NE, as shown in Fig 1C. (S2/S1 = 0.9+/−0.07 for capadenoson 6 · 10^−8^ M, n = 6, p = 0.1; S2/S1 = 0.6+/−0.05 for capadenoson 6 · 10^−7^ M, n = 5, p = 0.03; S2/S1 = 0.5+/−0.05 for CCPA 10^−6^ M, n = 7, p = 0.003).

Thus, we observed a reduction of stimulation-induced NE release by capadenoson at a dose of 6 · 10^−7^ M in SHR only. This reduction was comparable to the effect of 10^−6^ M CCPA.

### Effect of capadenoson on stress-induced increase of heart rate

In the *in vivo* experiments, Wistar rats and SHR were pre-treated with capadenoson at a concentration of 0.15 mg/kg for 5 days. On day 5, a stress test (physical restraint) was performed for 2 hours. The plasma concentration of capadenoson measured 3 hours after drug intake remained constant in the 5 days prior to the restraint stress test and averaged 7.63 µg/l on day 4 and 5, respectively.

**Figure 2:**
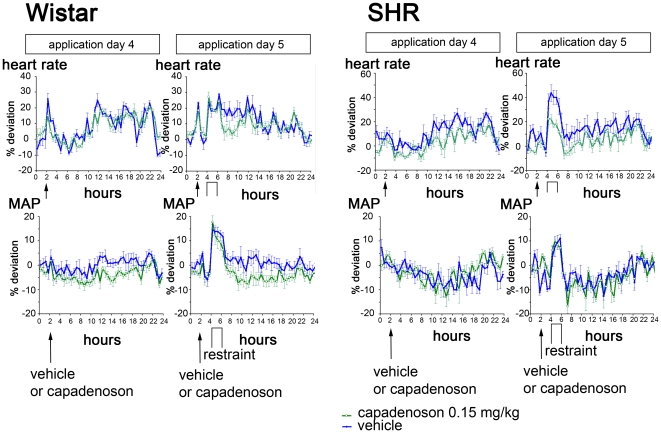
Telemetry data from Wistar and SHR during restraint experiment. Shown are the mean heart rate (as percent deviation from resting normal), and mean arterial pressure the day before, and on the day of the experiment. Blue lines depict vehicle-treated animals, green lines capadenoson-treated animals (0.15 mg/kg). Shown on the left side are data from Wistar rats (heart rate, above; MAP, below), on the right side the corresponding values from SHR. Wistar: vehicle and capadenoson each n = 20. SHR: vehicle and capadenoson each n = 12.

As listed in Table 1, mean heart rate was not affected by capadenoson during resting condition, neither in Wistar nor in SHR. During the 2 hours of physical restraint, we observed an increased heart rate in both strains (Wistar and SHR), which remained elevated throughout the restraint period (Fig. 2). After release from restraint, the rats recovered slowly, and heart rate fell to normal levels within 24 hours. As can be seen in Figure 2 and Table 1, in Wistar rats there was no difference in the increase in heart rate between animals treated with vehicle and animals treated with capadenoson at a dose of 0.15 mg/kg (relative increase in heart rate during stress: 18+/−3% in vehicle-treated animals, 15+/−2% in capadenoson-treated animals). In contrast, in SHR we observed a profound blunting of the stress-induced heart rate increase compared to vehicle-treated animals (relative increase in heart rate during stress: 36+/−4% in vehicle-treated animals, 20+/−4% in capadenoson treated animals; p<0.05).

**Table 1:** Heart rate and blood pressure during physical restraint in Wistar rats and SHR.

Group		Heart rate (1/min)			MAP (mmHg)		
		baseline	stress	relative change (%)	baseline	stress	relative change (%)
Wistar	vehicle	372+/−10	432+/−8	18+/−3	90+/−2	104+/−1	16+/−2
	Capadenoson	374+/−8	427+/−5	15+/−2	100+/−1	116+/−2	16+/−2
SHR	vehicle	324+/−11	438+/−7	36+/−4	164+/−5	189+/−3	16+/−12
	Capadenoson	328+/−6	391+/−8*	20+/−4*	175+/−7	189+/−6	9+/−10

Values are means +/− SEM. n = 20 for Wistar groups, n = 12 for SHR groups. MAP mean arterial pressure.

*p = 0.001 for comparison of the absolute and relative differences of heart rate during restraint stress in capadenoson-treated vs vehicle-treated SHR.

During stress, MAP increased in all groups, without any statistical difference between animals treated with vehicle or capadenoson, either in SHR or in Wistar (Fig. 2 and Table 1). However, there was a tendency towards a smaller increase of blood pressure during stress in SHR treated with capadenoson. In SHR, blood pressure was already elevated before the stress experiment, as expected in these animals.

### Determination of partial adenosine-A_1_ agonism of capadenoson

**Figure 3:**
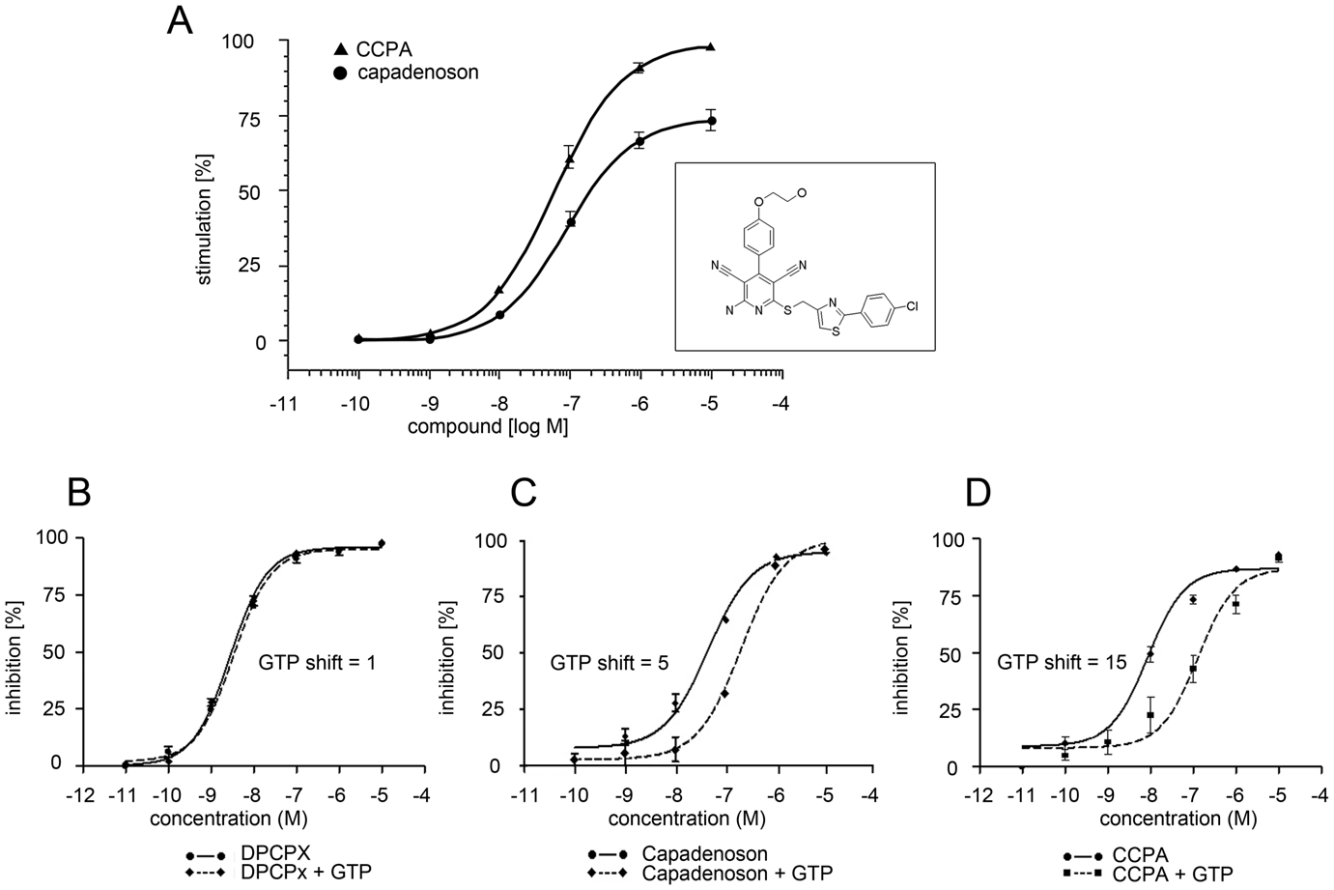
Capadenoson is a partial adenosine-A1 receptor agonist. A)Stimulation of [^35^S]GTPγS binding to human cortex membranes. Shown are means +/− SEM. B) GTP shift assay of the full antagonist DPCPX with and without addition of 1 mM GTP. C) GTP shift assay of the capadenoson, and D) of CCPA, with and without 1 mM GTP. Shown are means +/− SEM. Insert in panel A: Chemical structure of capadenoson.

Capadenoson is known to be a selective agonist of adenosine-A1 receptors [16]. To study the binding efficacy of capadenoson to A_1_ adenosine receptors, we measured ligand dependent G protein activation by binding of [^35^S]GTPγS to membranes of human brain cortex. CCPA, a well described full agonist of A_1_ adenosine receptors, was used as control to determine maximal [^35^S]GTPγS binding. As shown in Fig. 3A, capadenoson stimulated [^35^S]GTPγS binding only to 74±2% of the full effect of CCPA, suggesting partial agonism.

To further elucidate the pharmacological properties of capadenoson, GTP shift assays were performed with the standard full A1-agonist CCPA and the A1-antagonist 8-cyclopentyl-1,3-dipropylxanthine (DPCPX). CCPA showed a Ki value of 4.2 nM in the binding assay on rat cortical brain membranes (Fig. 3D). In the presence of 1 mM GTP this Ki value shifted to a value of 64 nM. Therefore the GTP shift for CCPA is 15. DPCPX showed a GTP shift of 1 with virtually identical Ki values in the absence and presence of GTP (Fig. 3B). Capadenson showed a Ki value of 24 nM in the binding assay. In the presence of 1 mM GTP this Ki value shifted to a value of 116 nM resulting in a GTP shift of 5 for Capadenoson (Fig. 3C). Therefore capadenoson could be demonstrated to be a partial agonist at the adenosine A1 receptor.

## Discussion

In the present study we could demonstrate that the partial adenosine A_1_-receptor agonist capadenoson leads to a profound reduction of stimulation-induced NE release in vitro as well as to blunted restraint stress-induced tachycardia in SHR, but not in Wistar rats. In a recent phase-II study of the same compound it was noted, that capadenoson specifically blunted the heart rate increase during exercise in a concentration dependent manner [13]. Together, these observations suggest that activation of presynaptic adenosine A_1_-receptors by a partial agonist may be a route to specifically dampen increases in heart rate caused by a high sympathetic tone.

Because the sympathetic nervous system has a major role in controlling heart rate, the present study was designed to evaluate the effect of capadenoson on the sympathetic tone in detail in an animal model of increased sympathetic activity. An established model in which an activated sympathetic tone plays a major role is the SHR strain, which develops hypertensive blood pressure levels at 5 to 6 weeks of age, followed by cardiac and vascular hypertrophy and ultimately end-organ failure later in life, if the hypertension remains untreated [24]. In SHR, an increased NE overflow from postganglionic nerve terminals has already been shown before [14], [15]. Thus, we chose SHR to study the effects of the novel adenosine-A_1_ agonist on the regulation of the sympathetic tone, and compared them to the effects in Wistar rats. We hypothesized, that activation of the cardiac adenosine-A_1_ receptor by capadenoson would reduce stimulation-induced NE release and consequently blunt the heart rate response during physical stress particularly in SHR.

As hypothesized, the stress-induced increase of the heart rate was significantly blunted by capadenoson in SHR. This *in vivo* effect of capadenoson was paralleled by a significant reduction of NE release upon stimulation in isolated hearts of SHR. The effects of adenosine agonists on norepinephrine release in Wistar rats have been characterized before. Burgdorf et al.[10] showed that activation of adenosine-A_1_ receptors by CCPA or the non-selective adenosine analogue R(-)N(6)-(2-phenylisopropyl)adenosine (R-PIA) decrease stimulation-induced NE release by about 50%, similar to the effect of CCPA we observed in the present study. Besides an activation of presynaptic A1-receptors, the attenuation of stimulation-induced norepinephrine release in the presence of desipramine as an uptake-1 inhibitor may also be explained by an activated extraneuronal uptake in SHR. [25] However, there is no evidence that capadenosone activates this extraneuronal uptake, whereas the presence of A1-receptors presynaptically is well described. It has been described that the mean concentration of norepinephrine is higher in the right ventricle and ventricular septum of SHR hearts compared with hearts of Wistar rats [26]. However, the functional meaning of the tissue concentration of norepinephrine is not clear, since the adrenergic activation may be more dependent on the release and turnover of norepinephrine as well as on the postsynaptic receptors than on the total amount of norepinephrine in the heart. Agonists of adenosine A_2_- and A_3_-receptor subtype seem to play no role in the modulation of cardiac NE release [27]. Adenosine has been shown to exert protective effects during ischemia and reperfusion through its potential to limit the release of norepinephrine[10], and is beneficial in preventing hypertrophic changes under catecholamine stimulation *in vitro* [28]. Thus, interventions aimed at a specific modulation of cardiac NE release would be highly promising in the treatment of various cardiac diseases, e.g. tachycardia-associated myocardial ischemia. Compared to a selective β_1_-blockade, a modulation of NE release would avoid the negative inotropic effects on the cardiomyocyte [29], [30].

In the present study we could demonstrate that capadenoson modulates NE release and stress-induced heart rate changes especially in SHR as a model for a high sympathetic tone, while heart rate in Wistar rats was not affected by capadenoson. Heart rate at rest was not affected in either strain. Such a selective attenuation of sympathetic activity as observed in the present study might be explained by the partial agonism of capadenoson on the adenosine-A_1_ receptor. The lack of effect of a partial agonist, as opposed to the action of a full agonist, on NE release in Wistar rats may be explained by the circumstance that the majority of the presynaptic A_1_ receptors are already activated by the endogenous full agonist adenosine, resulting in an effective feedback dampening of efferent sympathetic activity. By contrast, the presynaptic A_1_ receptors may be occupied to a much less extent by adenosine in SHR, which allows for the high sympathetic tone. Under these circumstances, a partial agonist such as capadenoson would be capable to activate unoccupied presynaptic adenosine A_1_ receptors during states of increased activity (e.g. during restraint stress or during stimulation in the *in vitro* experiments) at a significantly lower concentration than in Wistar rats. Other possible mechanisms underlying the strain-dependent actions of capadenoson on the stress response and NE release of SHR could be differences in the density or the molecular architecture of adenosine A1 receptors [31], or higher plasma concentrations of adenosine in SHR [32]. In a previous study, a similar effect of a different partial adenosine-A_1_ agonist, CVT-2759, was noted for the effects of adrenergic stimulation by isoproterenol in guinea pig myocytes. Similar to the present study, the partial adenosine A_1_ agonist had no effect on basal myocardial function, but attenuated isoproterenol-induced arrhythmias, also in a concentration-dependent manner [33]. Since only female rats were used, the results of the present study are limited to the female sex. Gender differences have been well described for the autonomic control of the cardiovascular system [34]. Therefore, future studies on the effect of capadenosone should taken into account the gender differences in sympathetic control.

In conclusion, we observed a selective reduction of stimulation-induced NE release by the partial adenosine A_1_-receptor agonist capadenoson in isolated SHR hearts as compared to hearts from Wistar rats. This effect translates into a blunted heart rate increase *in vivo* upon physical restraint in SHR during adenosine A_1_ agonism, whereas no effect was observed in Wistar rats. These results suggest that partial adenosine A_1_ agonism may be a new way to modulate the sympathetic control of cardiac function.

## Supporting Information

Figure S1 **Norepinephrine release after control stimulation in the absence of pharmacological intervention.** The second stimulation was performed 30 minutes after the first one without any pharmacological intervention apart from desipramine (10^−7^ M) for inhibition of neuronal uptake of norepinephrine. Shown is the ratio of norepinephrine overflow after a first (S1) and second (S2) stimulation. S2/S1 expressed as means +/− SEM, p = ns.
(TIF)

Figure S2 **Dose-response curves of capadenoson on mean arterial pressure.** A) Dose-response curves of 2 different concentrations in Wistar rats (n = 6 each), and controls. B) Dose-response curves of 4 different concentrations in SHR (n = 12 each), and controls.
(TIF)
