# Supplementary figures and images for: Selective Attenuation of Norepinephrine Release and Stress-Induced Heart Rate Increase by Partial Adenosine A1 Agonism
Source: PLoS One. 2011 Mar 28;6(3):e18048. doi: 10.1371/journal.pone.0018048 (PMC3065468; doi:10.1371/journal.pone.0018048)

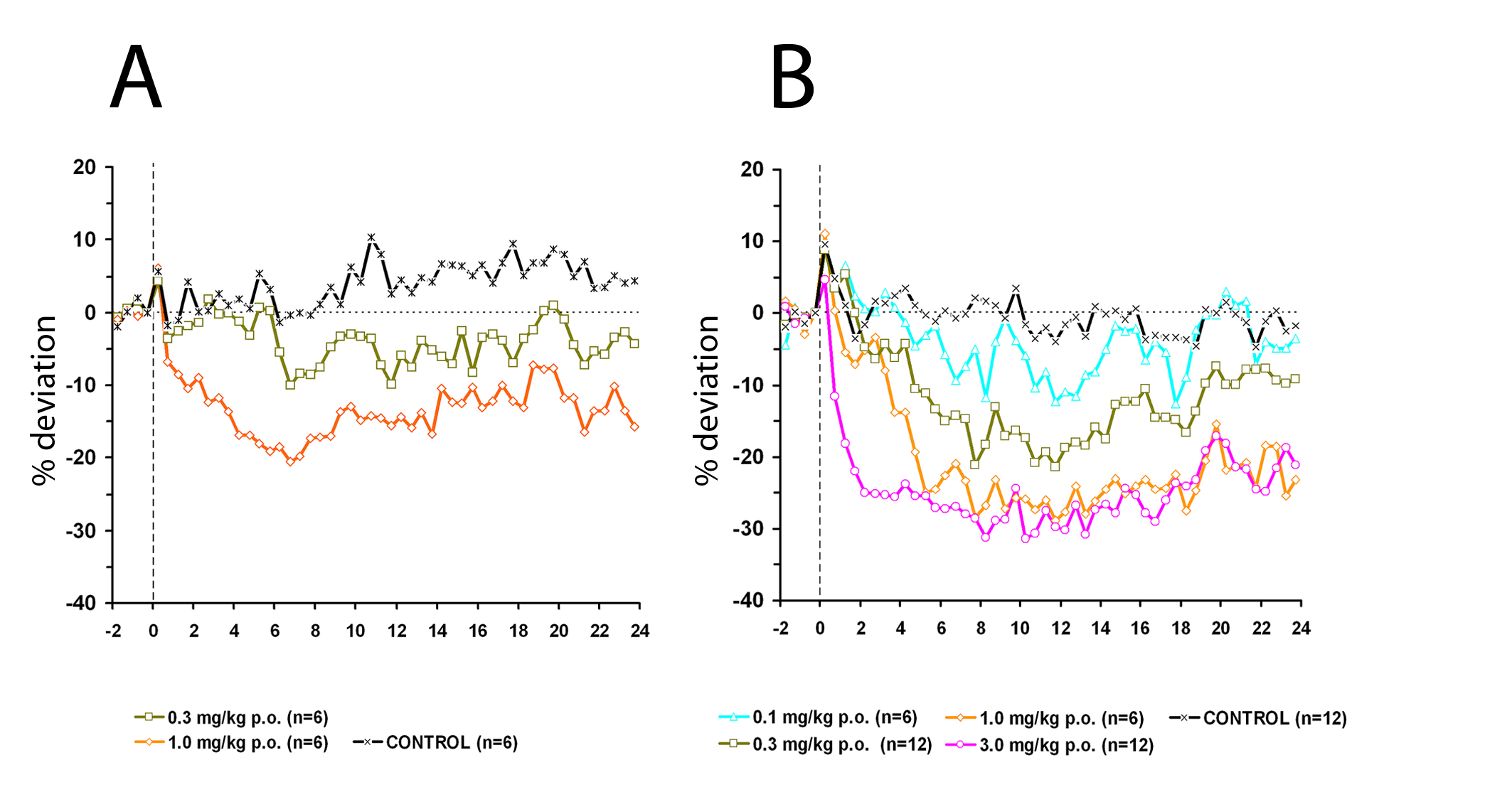

Supplement: Figure S1 — Norepinephrine release after control stimulation in the absence of pharmacological intervention. The second stimulation was performed 30 minutes after the first one without any pharmacological intervention apart from desipramine (10−7 M) for inhibition of neuronal uptake of norepinephrine. Shown is the ratio of norepinephrine overflow after a first (S1) and second (S2) stimulation. S2/S1 expressed as means +/− SEM, p = ns. (TIF) [file pone.0018048.s001.tif]

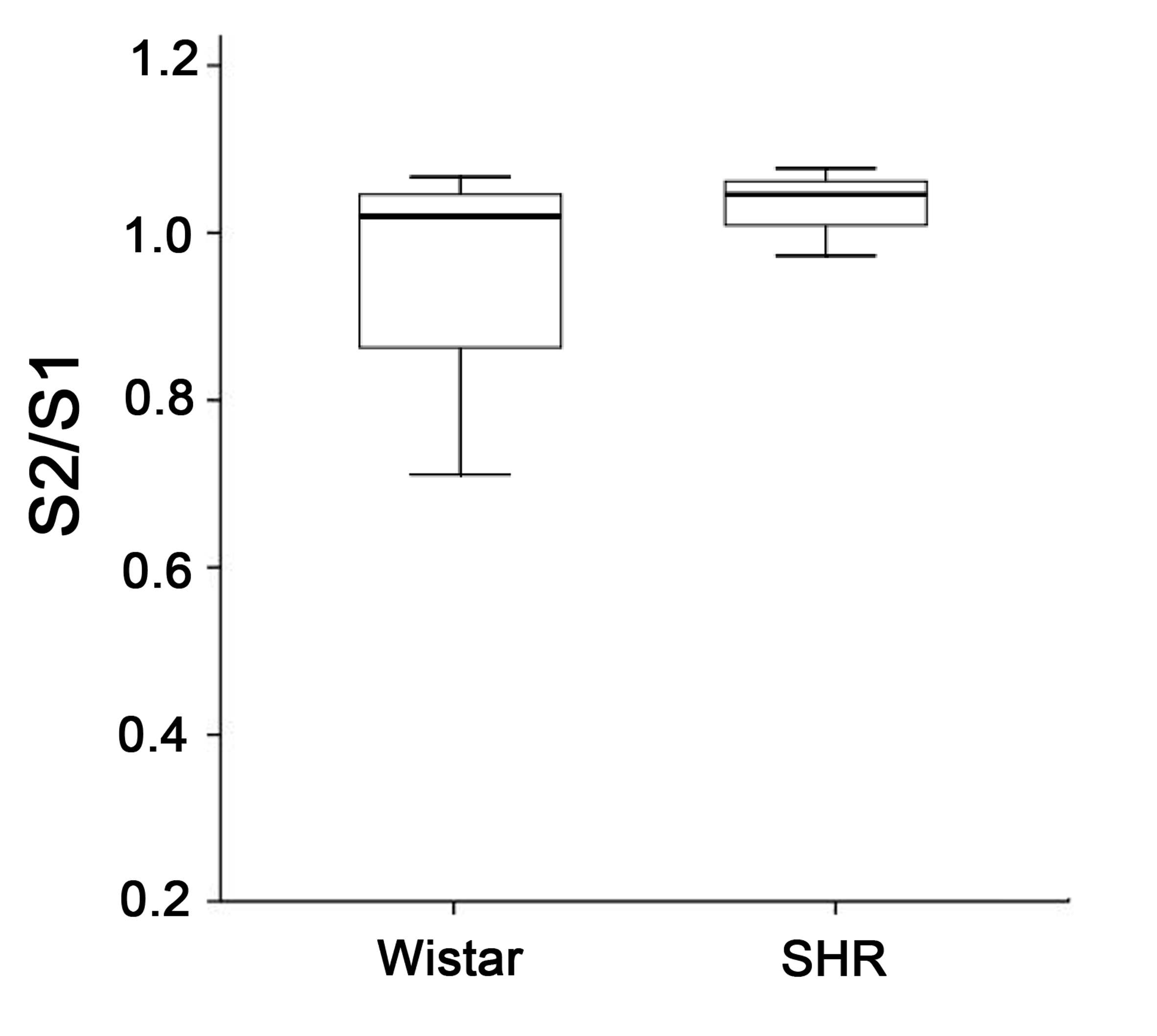

Supplement: Figure S2 — Dose-response curves of capadenoson on mean arterial pressure. A) Dose-response curves of 2 different concentrations in Wistar rats (n = 6 each), and controls. B) Dose-response curves of 4 different concentrations in SHR (n = 12 each), and controls. (TIF) [file pone.0018048.s002.tif]
